# First description of congenital toxoplasmosis after maternal coinfection with *Toxoplasma gondii* and severe acute respiratory syndrome coronavirus 2: a case report

**DOI:** 10.1186/s13256-023-03855-8

**Published:** 2023-04-04

**Authors:** Vu Thao-Vi Dao, Anastasia Anagnostou, Rolf Schlösser, Ulrich Rochwalsky, Uwe Groß, Sebastian Hoehl, Volkhard A. J. Kempf, Silke Besier

**Affiliations:** 1 Institute for Medical Microbiology and Infection Control, University Hospital, Goethe University, Paul-Ehrlich-Str. 40, 60596 Frankfurt am Main, Germany; 2 Department of Neonatology, University Hospital, Goethe University, Frankfurt am Main, Germany; 3 German Consulting Laboratory for Toxoplasma, Institute of Medical Microbiology and Virology, University Medical Center, Göttingen, Germany; 4 Institute for Medical Virology, University Hospital, Goethe University, Frankfurt am Main, Germany

**Keywords:** Congenital toxoplasmosis, SARS-CoV-2, Prematurity, Serological diagnosis, Case report

## Abstract

**Background:**

Congenital toxoplasmosis can be associated with serious clinical consequences from fetus to adulthood. Hence, early detection is required to minimize severe sequelae through appropriate therapy. We describe the first case of a congenital toxoplasmosis after maternal coinfection with *Toxoplasma gondii* and severe acute respiratory syndrome coronavirus 2 and the challenging serological diagnosis of the disease in this context.

**Case presentation:**

A Caucasian boy was born at 27 weeks 2 days of gestation by cesarean section due to maternal COVID-19-related respiratory failure. Postpartum serological screening of the mother revealed a previously unrecognized active *Toxoplasma gondii* infection. The premature child initially tested negative for anti- *Toxoplasma gondii* immunoglobulin A and M antibodies 1, 2 and 4 weeks after birth, whereas immunoglobulin G antibodies were only weakly positive with no evidence of child-specific production. Neither neurological nor ophthalmological abnormalities were detected. Approximately 3 months after birth, serological testing indicated a congenital toxoplasmosis by presence of immunoglobulin A and M, in combination with a child-specific immunoglobulin G synthesis. Additionally, cerebrospinal fluid was tested positive for *Toxoplasma gondii* DNA. Although no clinical manifestations of congenital toxoplasmosis were detected, an antiparasitic therapy was initiated to minimize the risk of late sequelae. There were no hints for a transplacental transmission of severe acute respiratory syndrome coronavirus 2.

**Conclusion:**

This case raises the awareness of possible coinfections with the risk of transplacental transmission in cases of maternal coronavirus disease 2019. The report emphasizes the need for screening vulnerable patients for toxoplasmosis in general and especially in the context of pregnancy. It becomes evident that prematurity can complicate the serological diagnosis of congenital toxoplasmosis due to a delayed antibody response. Repeated testing is recommended to carefully monitor children at risk and especially those with a history of preterm birth.

## Background

Up to two billion people worldwide are infected by the protozoan parasite *Toxoplasma gondii* (*T. gondii*) [1, 2]. Of these, approximately 190,100 cases per year account for congenital toxoplasmosis; and hence, this disease is considered by the World Health Organization (WHO) as a substantial burden with considerable sequelae [3]. Congenital infection occurs predominantly after primary maternal *T. gondii* infection; in rare cases maternofetal transmission has also been described after reactivation of a *T. gondii* infection during pregnancy, due to maternal immunosuppression [4, 5]. Clinical manifestations of the newborn (for example hydrocephalus, intracranial calcification, retinochoroiditis) depend on the time of infection during pregnancy. However, a significant number (14–85%) of infected children, without an apparent manifestation at birth, develop clinical symptoms months or years later [6]. Beyond these typical manifestations, developmental delay, deafness, epilepsy, or psychiatric disorders have been reported [7, 8]. Therefore, an undiagnosed congenital infection can cause serious sequelae [6]. To date, it is unknown whether the ongoing coronavirus disease 2019 (COVID-19) pandemic has any impact on the incidence, diagnosis, or manifestation of congenital toxoplasmosis. Here we describe for the first time a case of congenital toxoplasmosis that has been recorded after maternal *T. gondii* and severe acute respiratory syndrome coronavirus 2 (SARS CoV-2) coinfection.

## Case presentation

**Fig. 1:**
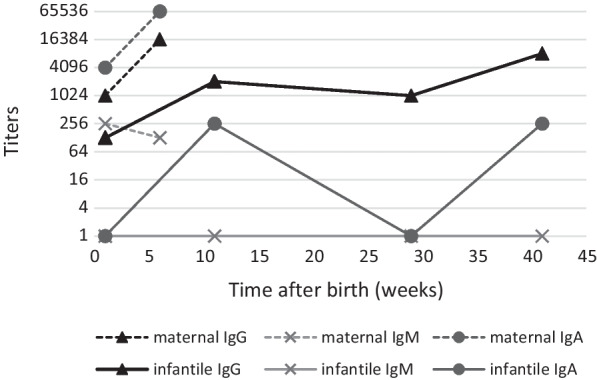
Time course of *T. gondii* antibody titers in mother’s and child’s sera. IgG and IgM were measured by indirect immunofluorescence testing (IFT; Euroimmun, Germany; titers < 16 are evaluated as negative). IgA was tested by immunosorbent agglutination assay [Ravo Diagnostika, Germany, titers < 16 (newborns) or < 64 (all other patients) are evaluated as negative]

**Fig. 2:**
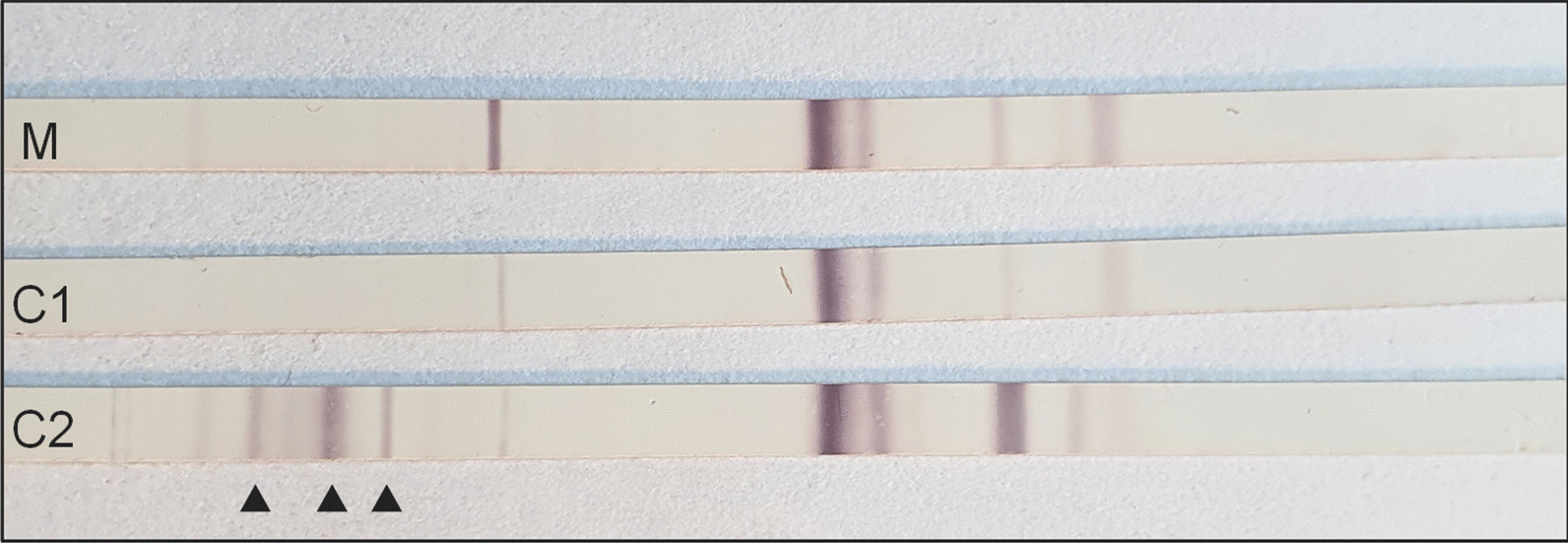
Comparative immunoblot analysis of mother (lane M) and child (lanes C1 and C2). The sera were taken 1 week (M, C1) and 11 weeks (C2) after birth. The arrows indicate the child’s neosynthesized antibodies

A 28-year-old gravida 1, para 0 woman of Caucasian ethnicity without relevant past medical history was admitted to the emergency ward at 26 weeks and 1 day of gestation because of progressive dyspnea due to SARS-CoV-2 pneumonia with the Alpha variant (B.1.1.7). Despite conventional therapy with supplemental oxygen, dexamethasone, and prophylactic treatment with ceftriaxone, increasing respiratory failure and impending need of endotracheal intubation forced the decision to primary cesarean section at 27 weeks and 2 days of gestation. The child’s apgar scores were 3, 5, and 6 at 1, 5, and 10 minutes, respectively. Due to prematurity, associated respiratory insufficiency, recurrent bradycardia, and infant respiratory distress syndrome, the boy of Caucasian ethnicity needed mechanical ventilation. First clinical examination after admission to the neonatal intensive care unit did not reveal any congenital malformations, except a secundum type atrial septal defect. Body temperature was initially elevated (38.0 °C), but inflammatory parameters remained normal [C-reactive protein (CRP) levels < 0.4 mg/dl]. The growth indices were below average with a birth weight of 980 g (10th–50th percentile), a head circumference of 25 cm (10th–50th percentile), and a length of 36 cm (10th–50th percentile). His clinical status stabilized in the further course and the boy was extubated on the 4th day of life. SARS-CoV-2 RNA was not detected in sequential respiratory and stool samples [polymerase chain reaction (PCR) targets: E-gene and ORF-region, limit of detection (LoD): about 50 genome equivalents/ml, Roche diagnostics, Switzerland]. Furthermore, SARS-CoV-2 immunoglobulin (Ig)M was not detected in a serum sample collected on the 3rd day of life, while SARS-CoV-2 antinucleocapside IgG was positive by chemiluminescent microparticle immunoassay [(CMIA), AdviseDx SARS-CoV-2 IgM und IgG test, Abbott Laboratories, USA]. An umbilical blood sample revealed a weak positive result for the E-gene target (cycle threshold value 36.83) and a negative result for the ORF-region target, which, considering the unremarkable follow-up samples, was not interpreted as evidence of a congenital SARS-CoV-2 infection. Surprisingly, postpartum maternal infection screening revealed a so far unknown *T. gondii* infection of the mother. Although the woman had no pet or similar exposure at home, serology clearly indicated a primary maternal infection during pregnancy, with proof of *Toxoplasma*-specific IgM antibodies and increasing titers of specific IgA and IgG antibodies (Fig. 1, Table 1). Due to a low IgG avidity (index 0.036) a maternal infection in the preceding 3–4 months could not be excluded. A PCR from maternal ethylenediamine tetraacetic acid (EDTA) blood was negative (target gene: 529 bp tandem repeat element, limit of detection: 4 *T. gondii* DNA copies per PCR reaction, Sacace Biotechnologies, Italy). Immediately after detection of the maternal *T. gondii* infection, serum of the premature child was analyzed. A low IgG titer was detected by indirect immunofluorescence testing (IFT), while IgM (IFT) and IgA [Enzyme-linked immunosorbent assay (ELISA), Immunosorbent agglutination assay (ISAGA)] tested negative (Fig. 1, Table 1). The competition test in a serum sample 1 week later was weakly positive, whereas selective screening for IgG, IgM [all enzyme-linked fluorescent immunoassay (ELFA)] and IgA antibodies (ELISA) against *T. gondii* remained surprisingly negative (Table 1). In addition, the IgG immunoblot profile (LDBIO Diagnostics, France) of mother and child was identical, suggesting a weak transfer of maternal IgG antibodies to the premature child (Fig. 2). Accordingly, a *T. gondii*-specific PCR from neonatal EDTA blood was negative. A follow-up serum sample 4 weeks after birth showed no significant serological changes (Table 1). Cerebral ultrasound, and ophthalmological and neurological examinations of the premature child were without any pathological findings. In addition, SARS-CoV-2 IgG had fallen below the cut-off value in a serum sample collected 5 weeks later, supporting the hypothesis of maternal IgG transfer. Six weeks after birth, the child was discharged from the intensive care unit. Unfortunately, the mother died 7 weeks after birth, despite maximum intensive care, including extracorporeal membrane oxygenation over a period of 6 weeks. Surprisingly, 11 weeks after birth, significant serological changes in the child’s serum were detected indicating congenital toxoplasmosis. At this timepoint, all *Toxoplasma*-specific antibody classes were detected to be clearly positive (Table 1). In addition, a significant increase of *T. gondii*-specific IgG antibody titers using the IFT was shown (1:128 versus 1:2048, Fig. 1). Accordingly, the IgG immunoblot profile of mother and child now indicated independent IgG synthesis by the child (Fig. 2). Immunoblots for IgA and IgM antibodies against *T. gondii* were negative, but IgA was confirmed to be positive by ISAGA. The child had not received any blood products so far. Moreover, a neonatal cerebrospinal fluid (CSF) sample revealed a positive *T. gondii*-PCR result (target gene: 529 bp tandem repeat element, limit of detection: 1–10 parasites per PCR reaction) and serum/liquor comparison blots for IgG indicated intrathecal IgG synthesis. CSF white blood cell count was 5/µl, glucose was 45 mg/dl, and protein was 1315 mg/l. Despite these pathologic CSF results, neither cerebral ultrasound nor magnetic resonance tomography (MRT) imaging indicated any cerebral abnormalities, and, moreover, neither ophthalmological nor clinical neurological manifestations were detected. Though the premature child appeared clinically asymptomatic, a *T. gondii*-specific treatment with pyrimethamine (1 mg/kg per day), sulfadiazine (50 mg/kg twice daily), and folinic acid (10 mg/week) was initiated to prevent or to minimize long-term sequelae of the *Toxoplasma* infection. The boy developed well and was discharged from hospital 4 months after birth. However, because of recurrent episodes of neutropenia, pyrimethamine had to be paused after 5 weeks. Dose increase of folinic acid only led to temporary improvement of neutropenia. Thus, sulfadiazine was additionally paused 11 weeks after start of therapy. No other antiparasitic therapy was initialized as the boy was still clinically asymptomatic and the serum control 29 weeks after birth was stable, with slightly descending IgG titers and absence of IgA (Fig. 1, Table 1). Ophthalmological, neurological, and pedaudiological examinations did not detect any abnormalities. Despite continuous folinic acid substitution, further episodes of neutropenia occurred. Thirteen weeks after stopping sulfadiazine treatment, a significant increase in IgG titers by IFT (1:1024 versus 1:8192), and occurrence of IgA by ISAGA, were observed (Fig. 1) suggesting a possible rebound phenomenon [9]. As neither cerebral MRT imaging nor standard control examinations revealed any pathological findings, it was decided to continue the concept of frequent serological, ophthalmological, neurological, and pedaudiological controls (every 3–6 months) without further antiparasitic therapy.

**Table 1:** *T. gondii* antibody screening results in the mother’s and child’s sera in time course

Serum	Time after birth (weeks)	IgG	IgM	IgA
		ELFA^a^ (U/ml)	ELFA^a^ (index)	ELISA^b^ (ratio)
Mother	1	Positive (23)	Positive (4.49)	Positive (2.78)
	6	Positive (260)	Positive (4.32)	Positive (> 5)
Child	1	–^c^	–^c^	Negative (0.03)
	2	Negative^d^ (< 4.0)	Negative^d^ (< 0.55)	Negative^d^ (< 0.8)
	4	Negative^d^ (< 4.0)	Negative^d^ (< 0.55)	Negative^d^ (< 0.8)
	11	Positive^d^ (46)	Positive^d^ (2.32)	Positive^d^ (6.97)
	29	Positive (95)	Negative (< 0.55)	Negative (0.14)
	41	Positive (> 300)	Negative (< 0.55)	Negative (0.22)

^a^Enzyme-linked fluorescent immunoassay , bioMérieux, France

^b^Enzyme-linked immunosorbent assay, Euroimmun, Germany

^c^This antibody class was only measured by indirect immunofluorescence testing because of a shortage of material (see Fig. 1)

^d^Results of the German consulting laboratory for *Toxoplasma* (Göttingen, Germany): ELFA, bioMérieux, France; ELISA, Biorad, USA

## Discussion

This case of a congenital toxoplasmosis after maternal *T. gondii* and SARS CoV-2 coinfection illustrates several important issues. First, it becomes evident that the serological diagnosis of congenital toxoplasmosis can be challenging in premature children and that repeated testing is necessary when initial results are negative. Generally, the presence of *Toxoplasma*-specific IgM and/or IgA in the serum of a neonate beyond the 1st week after birth proves congenital infection. This can be confirmed by detection of specific IgG antibodies produced by the child [10]. Also, an increase of the IgG titer in the 1st year of life, or the persistence of these antibodies, indicates a congenital infection, as maternally transferred IgG antibodies usually decline and disappear within 6–12 months [1, 4, 11]. In this report, the postnatal IgG level was surprisingly low and pathological antibody changes for IgM, IgA, and IgG were found approximately 3 months after birth. Indeed, it is known that neonatally performed *T. gondii* IgM and/or IgA tests can fail to identify cases of congenital toxoplasmosis in the first weeks of life, but most of these cases were associated with maternal antepartum *Toxoplasma*-specific treatment. In addition, *T. gondii* IgG titers can be reduced by antepartum, as well as by postnatal, *Toxoplasma*-specific treatment. It is assumed that the fetal/neonatal immune response is blocked or retarded by this treatment with a consequent delay in the production of serological markers [12]. As this point of maternal and/or postnatal treatment does not apply to the present case, the extreme prematurity of the child (birth < 28 weeks of gestation) most likely explains the initially low IgG level and the delayed antibody response. Studies regarding the maternofetal transport of immunoglobulins during human pregnancy suggest that the maternal IgG transfer begins in 13 weeks of gestation and increases until birth, with the largest amount of these antibodies being transferred in the third trimester. Hence, very premature children, as in this case, could have acquired little maternal IgG before delivery, which postnatally rapidly declines due to catabolism and blood dilution [13, 14]. In contrast, IgA and IgM do not cross the placenta. IgM production can start at 8 weeks of gestation [15], whereas first signs of IgA production are detectable earliest at 27 weeks of gestation [16], which was the time of the cesarean section in this report. In the literature, one case report addresses this possible association between prematurity and a delayed *T. gondii* IgM and IgA antibody response in the context of congenital toxoplasmosis, but in contrast to our case the mother was shortly treated with trimethoprim-sulfamethoxazole before the birth [17].

Besides prematurity, the time of maternal primary *T. gondii* infection during pregnancy could have influenced the neonatal antibody response. False-negative *T. gondii* IgM and IgA results have been described in cases of fetal infection very early or very late in gestation. In the context of an early fetal infection, the transient positive antibody response might have disappeared by the time of delivery, whereas in the context of a late fetal infection, initially negative *T. gondii* IgM and IgA results at birth could be attributable to delayed production of those antibodies [12]. In our case, the serological results with significantly rising maternal IgG and IgA titers, in combination with a stable IgM titer 6 weeks after birth, indicate a primary *T. gondii* infection of the mother in the late second trimester (Fig. 1). Thus, the short period between maternal infection and premature birth of the infected child at the end of the second trimester might be the most likely reason for the initial negative neonatal IgA and IgM test results. Moreover, it has been reported that antenatal corticosteroid treatment may be immunosuppressive for the premature child [18]. Hence, the antenatal maternal glucocorticoid therapy could also have influenced the child’s immune response.

Furthermore, this case demonstrates that one should be aware of coinfections with the risk of transplacental transmission in cases of maternal COVID-19. Despite the very severe course of maternal COVID-19, SARS CoV-2 itself was not vertically transmitted to the child. This is in line with several publications stating that transplacental transmission of SARS CoV-2 only rarely occurs [19]. However, it is known that pregnant women suffer from more severe courses of COVID-19 infections, with a higher risk of preterm birth [20]. Patients with severe disease in turn show profound immune dysregulation. Studies report that these patients had fewer natural killer (NK), CD3^+^, CD4^+^, and CD8^+^ T cells than those with non-severe disease, and that NK cells were found to be functionally exhausted. Additionally, a reduction in the circulating NK cell population has been described during pregnancy in this context [20]. However, an intact cellular immune system is necessary to control a *T. gondii* infection [4]. Thus, it could be hypothesized that the very severe maternal COVID-19 infection had negative effects on the infection control of the active *T. gondii* infection, maybe facilitating the transplacental transmission of tachyzoites in this case. In general, the risk of transplacental transmission of toxoplasmosis increases with duration of gestation at maternal seroconversion and is reported to be rather moderate in the second trimester, with a rate of approximately 40% at 26 weeks of gestation [21]. Further research is required to expand our understanding of SARS-CoV-2 and possible effects on the placental function and the growing fetus.

Finally, this report emphasizes the need for an increased diagnostic vigilance regarding maternal toxoplasmosis infection during pregnancy. A screening for *T. gondii* antibodies before or during pregnancy was not performed in this case, and the infection was only detected because of hospitalization of the mother due to the severe COVID-19 infection and the resulting premature birth of the child. As early diagnosis and treatment of acute maternal infections can lead to a reduction in the disease burden [22], preventive measures, including maternal screening for *T. gondii* antibodies, should be discussed as a general part of antenatal care.

## Conclusion

This report demonstrates that in cases of maternal SARS-CoV-2 infection, one should be aware of possible coinfections with the risk of transplacental transmission of the corresponding pathogens. It underlines the need for an increased diagnostic vigilance regarding toxoplasmosis infection in vulnerable patients and especially in the context of pregnancy and prematurity. Extremely preterm births can impede the serological diagnosis of congenital toxoplasmosis in terms of a delayed antibody response. Thus, it is recommended to monitor all children at risk, and particularly those with prematurity, by repeated *T. gondii* testing.

## Data Availability

The data used and analyzed during the current study are available from the corresponding author on reasonable request. The microbiological data are stored electronically in the laboratory information software (SWISSLAB, Nexus AG), and the clinical data are stored in the clinical information software (ORBIS, Dedalus HealthCare Systems Group) of the University Hospital of Goethe University, Frankfurt am Main, Germany.

## Abbreviations

CMIA: Chemiluminescent microparticle immunoassay
COVID-19: Coronavirus disease 2019
CRP: C-reactive protein DNA
CSF: Cerebrospinal fluid
DNA: Deoxyribonucleic acid
EDTA: Ethylenediamine tetraacetic acid
ELFA: Enzyme-linked fluorescent immunoassay
ELISA: Enzyme-linked immunosorbent assay
IFT: Indirect immunofluorescence testing
IgA: Immunoglobulin A
IgG: Immunoglobulin G
IgM: Immunoglobulin M
ISAGA: Immunosorbent agglutination assay
LoD: Limit of detection
PCR: Polymerase chain reaction
RNA: Ribonucleic acid
SARS-CoV-2: Severe acute respiratory syndrome coronavirus 2
*T. gondii*: *Toxoplasma gondii*
